# Draft genome sequence of *Colletotrichum sansevieriae* Sa-1–2, the anthracnose pathogen of *Sansevieria trifasciata*

**DOI:** 10.1016/j.dib.2018.03.083

**Published:** 2018-03-21

**Authors:** Masayuki Nakamura, Takashi Fujikawa, Daichi Nakamori, Hisashi Iwai

**Affiliations:** a Faculty of Agriculture, Kagoshima University, Kagoshima 890-0065, Japan; b Institute of Fruit Tree and Tea Science, National Agriculture and Food Research Organization (NARO), Fujimoto 2-1, Tsukuba, Ibaraki 305-8605, Japan

## Abstract

*Colletotrichum sansevieriae* is an ascomycete fungus causing anthracnose disease on plants in the genus *Sansevieria*. Here, we report the draft genome sequence of isolate Sa-1–2 of this fungus. The genome size is >51 Mb, and the assembly consists of 8647 contigs and contains 13,664 predicted protein-coding genes. Pathogenicity factors such as plant cell wall-degrading enzymes and effector proteins were also predicted. Additionally, the phylogenetic relationship of isolates from different *Colletotrichum* spp. was analyzed, revealing that the isolate belongs to a novel major clade consisting of species that infect succulent plants originating from Africa. The draft genome sequence has been deposited at GenBank under accession number NJHP00000000.

**Specifications Table**

**Table:** 

Subject area	*Biology*
More specific subject area	*Microbiology, Genomics*
Type of data	*Table, text file, figure*
How data was acquired	*Shotgun whole-genome DNA sequences using Ion PGM*
Data format	*Raw and assembled*
Experimental factors	*Genomic DNA extracted from Colletotrichum sansevieriae Sa-1–2*
Experimental features	*de novo assembly, gene prediction, phylogenetic analysis*
Data source location	*Kagoshima, Japan*
Data accessibility	*Deposited data is available at the National Center for Biotechnology*
	*Information (NCBI) under the accession number NJHP00000000* (https://www.ncbi.nlm.nih.gov/nuccore/NJHP00000000).

**Value of the data**

• The first draft genome sequence of *Colletotrichum sansevieriae*, a causal agent of anthracnose on sansevieria, is now available.
• Plant cell wall-degrading enzymes and effector proteins related to pathogenicity were predicted.
• The fungus belongs to a novel major clade in the genus *Colletotrichum*.
• These data will be useful for further research into the biology, evolution, and pathogenicity of anthracnose pathogens.

## 1 Data

*Colletotrichum sansevieriae* is an anthracnose pathogen identified as a new species in 2006 [1]. The fungus causes water-soaked lesions and leaf blight on sansevieria, which is one of the most important plants in subtropical regions in Japan as a potted ornamental and for cut leaves [1], [2]. The fungus shows pathogenicity only on *Sansevieria* spp., thereby having high host specificity. We are interested in learning which factors determine their host specificity. In this work, we performed draft genome analysis of the fungus to obtain fundamental genetic information for elucidating its host specificity determinants.

The draft assembly consists of 8647 contigs (GenBank accession number NJHP00000000) with a total length of 51.2 Mb, a G+C content of 50.8%, an N50 of 15,122 bp and an average length of 5922 bp. The total number of predicted tRNAs and rRNAs was 339 and 75, respectively. There were 13,664 predicted protein-coding genes, of which 1334 were classified as secreted.

The secretory proteins were annotated and 144 were predicted to be plant cell wall-degrading enzymes for cellulose, hemicellulose and pectin. Putative effector proteins were also analyzed, with a total of 316 proteins predicted, including 14 that had no match to sequences of other filamentous fungi deposited in GenBank. We compared the predicted effectors to those derived from other fungi [3] (Table 1). The percentage of effectors from isolate Sa-1–2 in the secretome was almost the same as for other hemibiotrophic fungi such as *C. higginsianum*, *Magnaporthe oryzae* and *Fusarium graminearum*. The obligate biotrophic fungus *Puccinia graminis* has the largest number of effector candidates in the secretome. In contrast, the necrotrophic fungus *Botrytis cinerea* has a smaller set of effector candidates.

**Table 1:** Effector prediction of *Colletotrichum sansevieriae* Sa-1–2 and comparison with various fungal species [3].

Fungal species	No. of predicted secretome	No. of predicted effectors
*C. sanevieriae* [This study]	1334	316 (23.6%)^a^
*C. higginsianum*	1528	471 (30.8%)
*C. graminicola*	1339	268 (20.0%)
*Puccinia graminis* f.sp. *tritici*	1946	846 (43.5%)
*Magnaporthe oryzae*	1576	485 (30.7)
*Botrytis cinerea*	933	183 (18.4%)

a Percentage of predicted effectors in the secretome.

*C. sansevieriae* was reported as a new species for the first time in 2006 [1] and was classified based only on the internal transcribed spacer 2 region of an rRNA gene. Thus, we also analyzed the fungus phylogenetically using the genetic data obtained in this work. Thus far, it has revealed that the genus *Colletotrichum* with straight conidia comprises five major clades [4]. Based on this work, *C. sansevieriae* Sa-1–2 belongs to a novel major clade consisting of newly identified species *C. euphorbiae* [5], *C. ledebouriae* [6] and *C. neosansevieriae* [7] that also infect succulent plants originating from Africa.

## 2 Experimental Design, Materials and Methods

### 2.1 Sequencing and assembly

Genomic DNA of the isolate Sa-1–2 was extracted from hyphae grown in potato dextrose broth (Difco, BD Diagnostic Systems, Sparks, MD, USA) and ultrasonicated by a Bioruptor (Cosmo Bio Co., Ltd., Tokyo, Japan). The genomic fragments were processed to template samples using the Ion Plus Fragment Library Kit (Thermo Fisher Scientific, Waltham, MA, USA) and the Ion PGM Hi-Q OT2 Kit with the Ion OneTouch 2 System (Thermo Fisher Scientific). Then, the template samples were sequenced using the Ion PGM Sequencing Hi-Q Kit (Thermo Fisher Scientific) and a 318 Chip with the next generation sequencer Ion PGM (Thermo Fisher Scientific). In total, 8,914,863 single reads with an average length of 247.35 bp were obtained. After these single reads were filtered with a Phred score cut-off of <20, the genome was *de novo* assembled using the CLC Genomics Workbench (Qiagen, Valencia, CA, USA).

### 2.2 Gene prediction

Protein coding genes were predicted with AUGUSTUS [8], [9] using *Fusarium graminearum* and *Magnaporthe oryzae* as species parameters and annotated with FunctionAnnotator [10] and BlastKOALA [11]. Secretory proteins were classified using SignalP 4.1 [12] and then used to predict effector proteins through EffectorP [13]. Prediction of RNA-encoding genes was performed for tRNAs using tRNAScan-SE [14] and for rRNAs using HMMER 3.0 [15].

### 2.3 Phylogenetic analysis

**Fig. 1:**
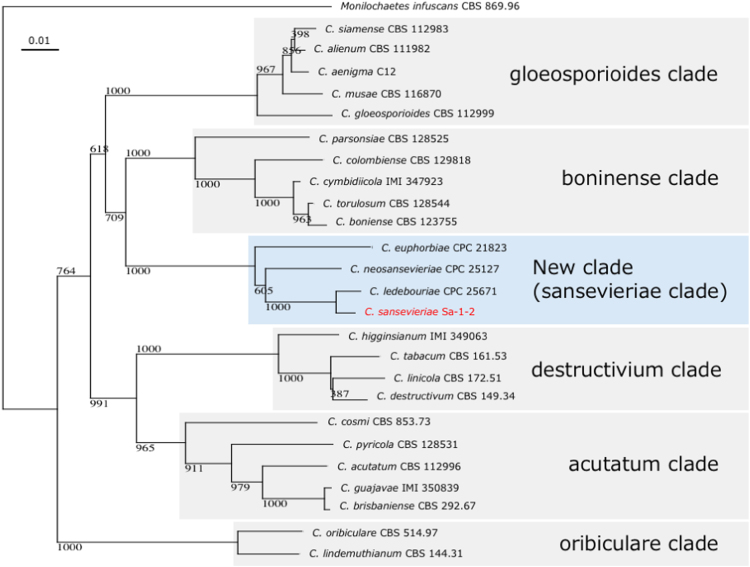
Phylogenetic tree derived from the neighbor-joining method of concatenated alignment of rRNA genes, histone H3 genes and chitin synthase genes. The bootstrap value of 1000 replications is given for each node. Source accession numbers are described after the scientific names.

The rRNA, histone H3 and chitin synthase genes of *C. sansevieriae* Sa-1–2 were aligned with other *Colletotrichum* spp. sequences deposited in the DDBJ/EMBL/GenBank database using ClustalX 2.0.5 [16]. A phylogenetic tree was constructed using ClustalX 2.0.5 and viewed by NJ plot [17] (Fig. 1).
